# Variant of PAI-2 gene is associated with coronary artery disease and recurrent coronary event risk in Chinese Han population

**DOI:** 10.1186/s12944-015-0150-y

**Published:** 2015-11-16

**Authors:** Xia Li, Jun-Yi Luo, Lei Zhang, Yi-Ning Yang, Xiang Xie, Fen Liu, Bang-Dang Chen, Yi-Tong Ma

**Affiliations:** Department of Geriatrics, Fifth Affiliated Hospital of Xinjiang Medical University, Urumqi, Xinjiang 830011 China; Department of Cardiology, First Affiliated Hospital of Xinjiang Medical University, Urumqi, Xinjiang 830054 China; Xinjiang Key Laboratory of Cardiovascular Disease Research, Urumqi, Xinjiang 830054 China

**Keywords:** *PAI-2* gene, Polymorphism, Coronary artery disease, Recurrent coronary event risk

## Abstract

**Background:**

Plasminogen activator inhibitor −2 (PAI-2) is an important molecular that plays a crucial role in vascular homeostasis and constitutes a critical response mechanism to cardiovascular injury, such as atherosclerosis, coronary artery disease (CAD).

**Methods:**

The aim of the current study was to explore the association between the variants in *PAI-2* gene and CAD and its prognoses. The three variants (rs8093048, rs9946657, rs9320032) of the *PAI-2* gene were detected in 407 patients with CAD and 518 control subjects. All patients with CAD underwent one-year follow-up for major adverse cardiac events (MACE).

**Results:**

The frequencies of the TT genotype and T allele of rs8093048 was significantly higher in CAD patients than that in control subjects (7.6 % vs.3.5 %, *P* = 0.003, 28.1 % vs.21.7 %, *P* < 0.001, respectively). Multifactor logistic regression analysis showed that the TT genotype of rs8093048 was a risk factor for CAD (OR = 1.455, 95 % CI: 1.069-1.980, *P* = 0.017). In addition, the follow-up data showed that CAD patients with rs8093048 TT genotype had significantly higher rate of refractory angina and MACE than those with CC or CT genotype (*P* = 0.032, *P* = 0.009, respectively). Cox regression analysis showed that rs8093048 TT genotype was the risk factor for the MACE (Hazard ratio = 5.672, 95 % CI = 1.992-16.152, *P* = 0.001).

**Conclusion:**

We firstly found that the variant of *PAI-2* gene was associated with CAD and recurrent coronary event risk in Chinese Han population, in Xinjiang.

## Background

The plasminogen activator (PA) system plays a key role in vascular homeostasis and constitutes a critical response mechanism to cardiovascular injury, such as atherosclerosis, coronary artery disease (CAD), myocardial infarction (MI) and restenosis [1]. The central components of the PA system are the proteolytic activators, urokinase-plasminogen activator (u-PA) and tissue-type plasminogen activator (t-PA), plasminogen (plg) and its degradation product, plasminogen activator inhibitor-1 (PAI-1) and plasminogen activator inhibitor −2 (PAI-2) [2]. PAI-2 is widely expressed in various cells, such as monocytes and macrophages, eosinophils, keratinocytes, microglia, and endothelial and epithelial cells, and is intensely upregulated in inflammation, infection, pregnancy, and other pathophysiological conditions [3–5]. It is predominantly expressed as a 47 kDa non-glycosylated intra cellular form located in the cytoplasm, but it can also be found as a glycosylated 60–70 kDa protein located in the extracellular surface [6, 7]. PAI-2 has been proposed to play a role in different processes, including protection of the retinoblastoma protein from degradation, regulation of keratinocyte and monocyte proliferation, and differentiation, priming interferon α/β responses, inhibition of annexin-1 cleavage, interleukin 1β processing, promotion of adipose tissue development, and the inhibition of apoptosis [8].

CAD is a complex and multifactorial disorder involving the interaction of genetic factors and environmental factors. Despite significant improvement in clinical management of the CAD, it is still reported as the most common cause of adult deaths worldwide [9, 10]. While lifestyle modification has reduced the mortality rate, the candidate gene approach has provided new insights for exploring diagnostic and therapeutic approaches. *PAI-2* gene consists of 8 exons spanning 16.18 kb on the long arm of chromosome 18 [11]. Nur Buyru et al. investigated 45 myocardial infarction patients and 20 control subjects, they found that the variant at the 413 position (AA genotype) of *PAI-2* gene was associated with an increased risk of MI [12]. James P. Corsetti et al. found that the polymorphism of rs6095 in *PAI-2* gene was the continued significant risk of recurrent coronary event among 166 patients with high high-density lipoprotein-cholesterol (HDL-C) and C-reactive protein levels [13]. Moreover, Zhao et al. genotyped 57 patients with CAD and 62 controls, it indicated that *PAI-2* gene Ser/Cys413 15588 G/C polymorphism was associated with CAD and the C allele was a risk factor for CAD [14]. In the current study, we recruited a larger sample (407 CAD patients and 518 control subjects) to investigate the association between *PAI-2* gene and CAD in Chinese Han population. And we built a prospective cohort study of the CAD patients to analyze the major adverse cardiac events (MACE) among different genotypes of PAI-2 gene for a mean period of one-year follow-up.

## Methods

### Ethics statement

Written informed consents were obtained from all the participants. The study was approved by the Ethics Committee of the First Affiliated Hospital of Xinjiang Medical University and conducted according to the principles outlined in the Declaration of Helsinki.

### Subjects

All CAD patients and control subjects were recruited at the First Affiliated Hospital of Xinjiang Medical University from 2010 to 2013. 407 CAD patients and 518control subjects were randomly recruited in this study. All of the participants were genetically-unrelated. All of the CAD patients were defined by angiographic means (main coronary artery stenosis of >50 %). For the control group, we selected healthy participants matched for sex, and age. Control subjects were selected from the Cardiovascular Risk Survey (CRS) [15, 16]. Briefly, the CRS is a prospective, observational cohort study designed to investigate the prevalence, incidence, and risk factors for cardiovascular disease in the Han, Uygur, and Kazakh populations in Xinjiang of China. The control participants were defined as with no coronary vessel stenosis or clinical and electrocardiographic evidence of MI or CAD. All patients with kidney disease, malignancy, connective tissue disease, schizophrenia, or chronic inflammatory disease were excluded.

### Laboratory examination and definition of cardiovascular risk factors

The plasma concentration of blood triglyceride (TG), total cholesterol (TC), HDL-C, and low-density lipoprotein-cholesterol (LDL-C), were measured using standard methods in the Clinical Laboratory Department of First Affiliated Hospital, Xinjiang Medical University. Hypertension was defined as history of hypertension and/or an average systolic blood pressure (SBP) ≥ 140 mmHg and/or an average diastolic blood pressure (DBP) ≥90 mmHg on at least 2 separate occasions according to the medical examination and history. Diabetes was defined as history or presence of diabetes and/or a fasting plasma glucose level >7.0 mmol/L (126 mg/dl) on 2 separate occasions, or a random glucose value of >11.1 mmol/L (200 mg/dl) on ≥1 occasion. Body mass index (BMI) was calculated from standardized measurements of height and weight. Persons reporting regular tobacco use in the previous 6 months were considered as current smokers.

### DNA Extraction

Blood samples were collected from all participants after fasting for 12 h. Genome DNA was extracted from peripheral vein blood leukocytes using a whole blood genome extraction kit (Boiteke Corporation, Beijing, China).

### SNP selection

In this study, we screened the data on the National Center for Biotechnology Information SNP database (www.ncbi.nlm.nih.gov/SNP) for the SNPs of *PAI-2* gene. There are 141 SNPs listed in the SNP database. We used the minor allele frequency (MAF) ≥0.1 and linkage disequilibrium patterns with r^2^ ≥ 0.5 as a cut off by the Haploview 4.2 software. We achieved 7 tag SNPs listed in the HapMap phrase II database. SNPs with relatively high MAF have been shown to be useful as genetic markers in genetic association studies. Based on the screening for markers to be used in our genetic association research, we selected 3 SNPs (rs9946657, rs8093048, rs9320032) which located in a haplotype region. rs8093048 located in the 3’untranslated region (UTR) of the flotillin-2 gene, rs9320032 located in the intron, and rs9946657 located in the 5’ UTR.

### Genotyping

We used TaqMan® SNP genotyping assays (Applied Biosystems, Foster City, CA). The primers and probes used in the TaqMan® SNP Genotyping Assays were chosen based on information available at the ABI website. The polymerase chain reaction (PCR) amplification was performed in 96 well plates adding 2.5 μL of TaqMan Universal Master Mix (40×), 0.15 μL probes and 1.85 μL ddH2O in a 6 μL final reaction volume containing 1.5 μL DNA. Separate negative control group and positive control group in each plate. The PCR amplification was performed using the 7900HT sequence detection system and the amplified condition was as follows: 95 °C for 5 min; 40 cycles of 95 °C for 15 s; and 60 °C for 1 min. All 96 well plates were read on Sequence Detection Systems (SDS) automation controller software v2.3 (ABI).

### Clinical follow-up and judgment of endpoint events

Follow-up examination was performed on one year after discharge. Follow-up information was collected through out-patient review, telephone call, and readmission. Record the general information of the patients and the end point events. The primary end point used was MACE, defined as death, refractory angina, MI, target vessel revascularization (TVR), and cerebrovascular events [transient ischemic attack (TIA), stroke and reversible ischemic injury].

### Statistical analysis

All analyses were performed using the computer software Statistical Package for Social Sciences-SPSS for Windows (version 17.0). Hardy-Weinberg equilibrium was assessed by using chi-square analysis. Continuous data were expressed as mean ± standard deviation (SD) and the differences between the CAD patients and the control participants were assessed by independent samples *t* test. Categorical data and distribution of genotypes and alleles were shown as percentages (%) and the differences between the two groups were assessed by chi-square test. Logistic regression analysis was performed to assess the contribution of the major risk factor to CAD and the value was shown as odds ratio (OR) and its 95 % confidence interval (CI). In addition, the COX regressive analysis was used to assess the clinical prognosis among different genotype of Flotillin-2 gene. *P* < 0.05 was considered to indicate a statistically significant difference.

## Results

### Characteristics of participants

A total of 925 individuals (407 patients with CAD and 518 healthy controls) were participated in this study. There were no differences in age between CAD patients and control subjects, suggesting that the study was an age‑matched case‑control study. The concentration of HDL-C was significantly lower in patients with CAD than that in control subjects (*P* = 0.026). However, the percentage of individuals with EH, DM and smoking were significantly higher in CAD patients than that in control subjects (*P* = 0.013, *P* < 0.001, and *P* < 0.001, respectively). No significant differences in BMI, pulse and concentration of TC, TG, LDL-C were observed between the two groups (Table 1).

**Table 1:** Characteristics of study participants in Chinese Han population

	CAD patients	Control subjects	*P* value
Number of subjects	407	518	
Age(years)	62.36(10.62)	62.40(9.45)	0.951
Men, n (%)	290(71.3)	348(67.2)	0.184
BMI (kg/m^2^)	25.70(3.38)	26.06(4.10)	0.153
Pulse (beats/min)	73.04(10.42)	72.72(10.32)	0.641
TC (mmol/L)	4.44(0.97)	4.33(1.03)	0.165
TG (mmol/L)	1.84(1.23)	1.91(1.62)	0.470
LDL (mmol/L)	2.46(0.91)	2.44(0.84)	0.824
HDL (mmol/L)	1.16(0.49)	1.24(0.58)	0.026*
EH,n (%)	219(53.8)	236(45.6)	0.013*
DM, n (%)	107(26.3)	61(11.8)	<0.001*
Smoking, n (%)	233(57.2)	184(35.5)	<0.001*

Continuous variables are expressed as mean ± s.d. Categorical variables are expressed as percentages. *CAD* coronary artery disease, *BMI* body mass index, *total TC* cholesterol, *TG* triglycerides, *LDL-C* Low-density lipoprotein-cholesterol, *HDL-C* high-density lipoprotein-cholesterol, *EH* essential hypertension, *DM* diabetes mellitus. The *P* value of the continuous variables was calculated by the independent samples *t* test. The *P* value of the categorical variables was calculated by Chi square test. *P < 0.05

### Distribution of the flotillin-2 genotype

The genotype distribution of the three SNPs in *PAI-2* gene did not show any significant difference from Hardy-Weinberg equilibrium (all *P* > 0.05, the data did not show). The frequency of genotype and allele of *PAI-2* gene in CAD patients and controls were shown in Table 2. There were significant differences in genotypic and allelic distribution of *PAI-2* gene between CAD patients and controls. The *PAI-2* gene rs8093048 TT genotype and T allele distribution was significantly higher in CAD patients than that in control subjects (7.6 % versus 3.5 %, *P* = 0.003, 28.1 % versus 21.7 %, *P* < 0.001, respectively). For rs9946657 and rs9320032, we did not found any significant difference in the genotypic and allelic distribution of *PAI-2* gene between CAD patients and controls.

**Table 2:** Genotype and allele distributions of PAI-2 gene in CAD patients and controls

Variants	Genotyping/allele	CAD patients	Control subjects	*P* value
rs9946657	GG	114(28.0 %)	155(29.9 %)	
	GT	211(51.8 %)	273(52.7 %)	
	TT	82(20.2 %)	90(17.4 %)	0.532
	T	375(46.1 %)	453(43.7 %)	
	G	439(53.9)	583(56.3 %)	0.314
rs8093048	CC	209(51.4 %)	311(60.0 %)	
	CT	167(41.0 %)	189(36.5 %)	
	TT	31(7.6 %)	18(3.5 %)	0.003
	C	585(71.9 %)	811(78.3 %)	
	T	229(28.1 %)	225(21.7 %)	<0.001*
rs9320032	CC	13(3.2 %)	18(3.5 %)	
	CT	119(29.2 %)	152(29.3 %)	
	TT	275(67.6 %)	348(67.2 %)	0.828
	C	145(17.8 %)	188(18.1 %)	
	T	669(82.2 %)	848(81.9 %)	0.853

### Logistic regression analysis of CAD risk factors

Multifactor logistic regression analyzed the contribution of the major risk factor to CAD. It showed that rs8093048 T allele carriers (TT + CT), DM, and smoking were the risk factors for CAD. Following adjustments for DM, EH, TC, LDL-C, HDL-C, and smoking, the subjects with T allele of rs8093048 (TT + CT) had a significantly higher risk of CAD (OR = 1.455, 95 % CI: 1.069-1.980, *P* = 0.017) (Table 3).

**Table 3:** Multiple logistic regression analysis for CAD patients and control subjects in Chinese Han population

Risk factors	Odd ratios	95 % CI	*P* value
rs8093048 (TT + CT v.s. CC)	1.455	1.069-1.980	0.017*
DM	1.724	1.174-2.531	0.005*
Hypertension	1.263	0.925-1.725	0.142
TC	1.165	0.951-1.429	0.141
LDL	0.916	0.726-1.155	0.457
HDL	0.805	0.60-1.079	0.146
Smoking	3.506	2.564-4.794	<0.001*

*CAD* coronary artery disease, *DM* diabetes mellitus, *LDL-C* Low-density lipoprotein-cholesterol, *HDL-C* high-density lipoprotein-cholesterol. *P < 0.05

### Result of clinical follow-up

**Fig. 1:**
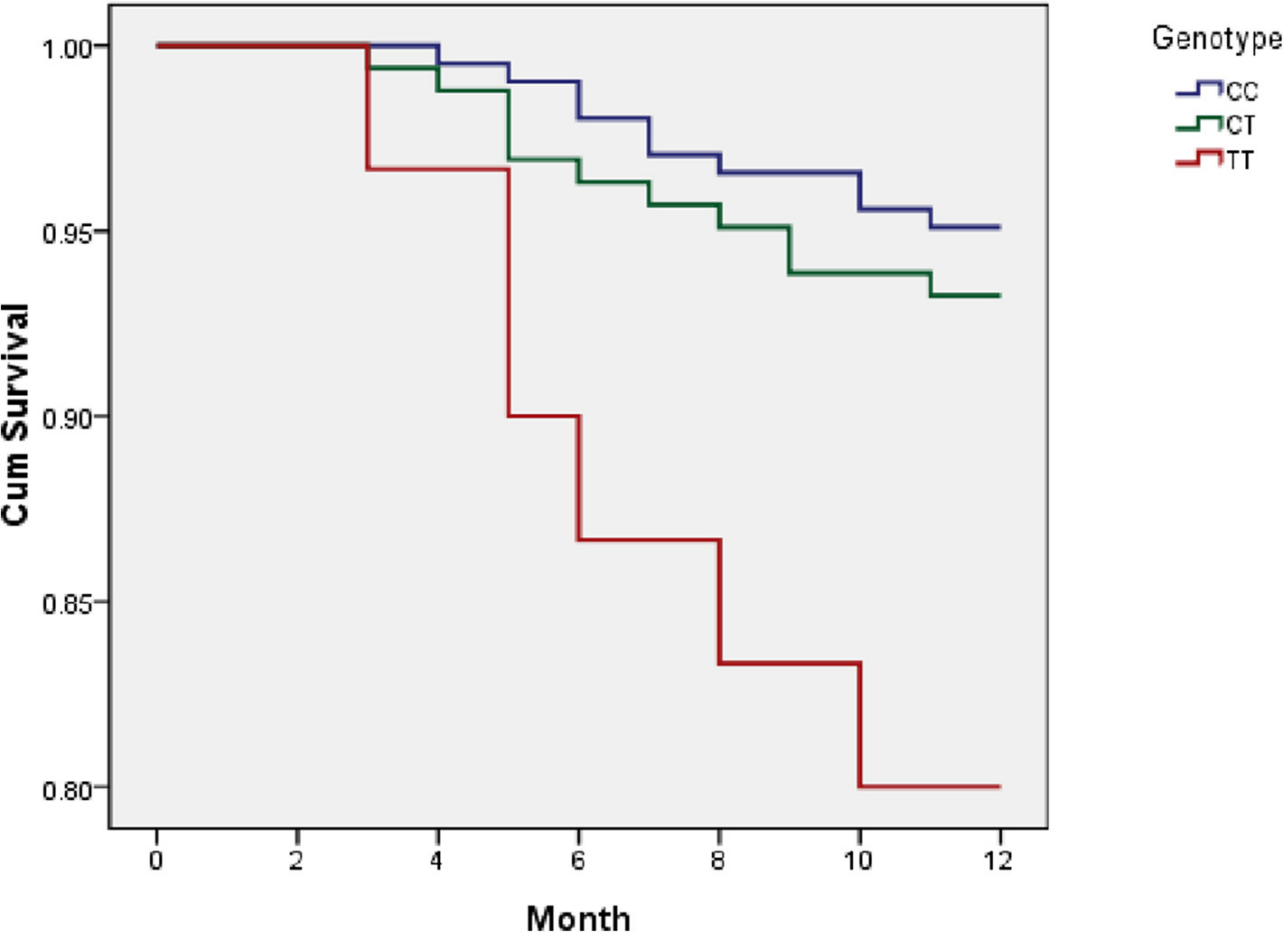
The survival among different genotype of rs8093048 in Chinese Han CAD patients

One year follow-up data were available in 397 of the original 407 CAD patients (Table 4). The missing rate was 2.46 %. Overall, 27 MACE occurred during the one-year study (6.8 %). There were 4 patients died (1.0 %), 6 patients occurred refractory angina (1.5 %), 5 patients had MI (1.25 %), 4 patients had target vessel revascularization (1.0 %), 8 patients occurred cerebrovascular events (2.0 %). The rate of refractory angina and MACE were significantly different among the three different genotype of rs8093048 (Fig. 1). Cox regression analysis showed that rs8093048 TT genotype was the risk factor for the MACE (Hazard ratio = 5.672, 95 % CI = 1.992-16.152, *P* = 0.001) (Table 5).

**Table 4:** The occurrence of adverse events in patients with CAD during one year follow-up

End point	CC(204)	CT(163)	TT(30)	*P* value
Death, n (%)	2(1.0)	1(0.6)	1(3.3)	0.39
refractory angina, n (%)	1(0.5)	3(1.8)	2(6.7)	0.032*
MI, n (%)	2(1.0)	2(1.2)	1(3.3)	0.558
TVR, n (%)	1(0.5)	2(1.2)	1(3.3)	0.324
cerebrovascular events, n (%)	4(2.0)	3(1.8)	1(3.3)	0.864
MACE, n (%)	10(4.9)	11(6.7)	6(20.0)	0.009*

*MI* myocardial infarction, *TVR* target vessel revascularization. *P < 0.05

**Table 5:** COX regression analysis in Chinese Han CAD patients

	*OR*	SE	*P* value	Hazard ratio(95 % *CI*)
BMI	0.04	0.061	0.514	1.041(0.923-1.174)
TG	0.069	0.174	0.694	1.071(0.761-1.507)
TC	−0.054	0.257	0.833	0.947(0.573-1.567)
LDL-C	0.328	0.252	0.193	1.388(0.847-2.274)
HDL-C	0.123	0.384	0.749	1.131(0.533-2.399)
Hypertension	0.06	0.413	0.884	1.062(0.473-2.387)
DM	0.647	0.415	0.119	1.909(0.847-4.304)
Smoking	0.274	0.442	0.536	1.315(0.552-3.128)
rs8093048 genotype				
CC	-	-	-	-
CT	0.38	0.445	0.393	1.463(0.611-3.501)
TT	1.736	0.534	0.001*	5.672(1.992-16.152)

*BMI* body mass index, *TC* cholesterol, *TG* triglycerides, *LDL-C* Low-density lipoprotein-cholesterol, *HDL-C* high-density lipoprotein-cholesterol, *DM* diabetes mellitus. *P < 0.05

## Discussion

In the current study, we observed that the variation in the *PAI-2* gene was associated with CAD and its prognosis in Chinese Han subjects. Individuals with TT genotype of rs8093048 had significantly higher risk of CAD and worse prognosis than that with CC or CT genotype. The rate of refractory angina was significantly higher in CAD patients with TT genotype than that with CC or CT genotype. The results demonstrated that the polymorphism of *PAI-2* gene may be as a marker of CAD and its prognosis.

PAI-2 is an important molecular of the serpin superfamily. It’s an efficient inhibitor of t-PA and u-PA and an important regulatory element in fibrinolysis. To maintain normal blood flow, fibrinolysis and hemostasis should exist in a precise balance in which fibrinolysis restricts excessive blood clots and hemostasis prevents excessive blood loss. Because serine proteases in both fibrinolysis and hemostasis are the key enzymes, both processes are primarily controlled by serine protease inhibitors (serpins, such as PAI-1, PAI-2, α2-AP) [17–20]. Genetic deficiency, excessive expression, or dysfunction of one or more of these serpins may cause pathological bleeding or thrombosis. In normal physiological conditions, PAI-2 is only detectable in plasma during pregnancy and probably has a role in maintaining the placenta or in embryonic development [21, 22]. It’s worth noting that there was strong correlation between elevated tPA and PAI-2 levels in gingival crevicular fluid during inflammation, and the balance between them remains constant during the inflammatory process [23, 24]. Moreover, the overexpression of PAI-2 could inhibit the proliferation and migration and promotes the apoptosis of human pulmonary arterial smooth muscle cells [25]. Intracellular PAI-2 affects cellular proliferation and differentiation, alters gene expression, and inhibits apoptosis [26, 27].

But so far, the reason and mechanism of PAI-2 and CAD are unclear. We firstly indicated that *PAI-2* gene may be a candidate gene of CAD in Chinese Han population. It was consistent with the previous researches. Cerrahpasa et al. found that AA genotype of the *PAI-2* gene was found to be more frequent among those subjects with MI [12]. James et al. demonstrated independent association of the *PAI-2* polymorphism reference allele with recurrent cardiovascular disease risk [13]. Zhao et al. reported that *PAI-2* gene Ser/Cys413 polymorphism is associated with CAD, the C allele is a risk factor for CAD and the G allele is a protective factor for CAD [14]. However, the Physician’s Health Study and ECTIM did not support an association between polymorphism *PAI-2* and acute coronary syndrome [17]. The contradiction may lie in the differences in gene expression between different ethnic population and the small >sample in the two studies. In the other hand, Palafox-Sa´nchez found that the functional Ser(413)/Ser(413) *PAI-2* polymorphism was associated with susceptibility and damage index score in systemic lupus erythematosus [28]. Moreover, the polymorphism of *PAI-2* gene was also associated with antiphospholipid syndrome [29]. These disease states all relate to disorders in hemostasis, highlighting the role of *PAI-2* gene in this system.

There were certain limitations in our study. Firstly, we only detected the association between the 3 SNPs in *PAI-2* gene with the CAD. As we know, SNPs are the most abundant form of genetic variations and have a great potential for mapping genes underlying complex genetic traits. But their effects were micro-effect. Much more studies of the other SNPs are needed to investigate the mechanism of CAD and *PAI-2* gene. Secondly, the level of plasma concentration of PAI-2 was not measured in our study because of their low concentrations. However, there was an advantage in the present study what the sample size was relatively large. It was better to indicate the association between *PAI-2* gene and CAD.

## Conclusions

In conclusion, we are more convinced that the variant in *PAI-2* gene may be associated with the susceptibility to CAD and its prognosis in Chinese Han population. Individuals with rs8093048 TT and CT genotype had significantly higher risk of CAD and worse prognosis than that with CC genotype. The rate of refractory angina was significantly higher in CAD patients with TT genotype than that with CC or CT genotype.
